# Genome-Wide Association Study between Single Nucleotide Polymorphisms and Flight Speed in Nellore Cattle

**DOI:** 10.1371/journal.pone.0156956

**Published:** 2016-06-14

**Authors:** Tiago Silva Valente, Fernando Baldi, Aline Cristina Sant’Anna, Lucia Galvão Albuquerque, Mateus José Rodrigues Paranhos da Costa

**Affiliations:** 1 Universidade Estadual Paulista (Unesp), Faculdade de Ciências Agrárias e Veterinárias, Departamento de Zootecnia, Via de Acesso Professor Paulo Donato Castellane, Jaboticabal, SP 14.884-900, Brazil; 2 Universidade Federal de Juiz de Fora (UFJF), Instituto de Ciências Biológicas, Departamento de Zoologia, Rua José Lourenço Kelmer, Juiz de Fora, MG 36.036-900, Brazil; CSIRO, AUSTRALIA

## Abstract

**Introduction:**

Cattle temperament is an important factor that affects the profitability of beef cattle enterprises, due to its relationship with productivity traits, animal welfare and labor safety. Temperament is a complex phenotype often assessed by measuring a series of behavioral traits, which result from the effects of multiple environmental and genetic factors, and their interactions. The aims of this study were to perform a genome-wide association study and detect genomic regions, potential candidate genes and their biological mechanisms underlying temperament, measured by flight speed (FS) test in Nellore cattle.

**Materials and Methods:**

The genome-wide association study (GWAS) was performed using a single-step procedure (ssGBLUP) which combined simultaneously all 16,600 phenotypes from genotyped and non-genotyped animals, full pedigree information of 162,645 animals and 1,384 genotyped animals in one step. The animals were genotyped with High Density Bovine SNP BeadChip which contains 777,962 SNP markers. After quality control (QC) a total of 455,374 SNPs remained.

**Results:**

Heritability estimated for FS was 0.21 ± 0.02. Consecutive SNPs explaining 1% or more of the total additive genetic variance were considered as windows associated with FS. Nine candidate regions located on eight different *Bos taurus* chromosomes (BTA) (1 at 73 Mb, 2 at 65 Mb, 5 at 22 Mb and 119 Mb, 9 at 98 Mb, 11 at 67 Mb, 15 at 16 Mb, 17 at 63 Kb, and 26 at 47 Mb) were identified. The candidate genes identified in these regions were *NCKAP5* (BTA2), *PARK2* (BTA9), *ANTXR1* (BTA11), *GUCY1A2* (BTA15), *CPE* (BTA17) and *DOCK1* (BTA26). Among these genes *PARK2*, *GUCY1A2*, *CPE* and *DOCK1* are related to dopaminergic system, memory formation, biosynthesis of peptide hormone and neurotransmitter and brain development, respectively.

**Conclusions:**

Our findings allowed us to identify nine genomic regions (SNP windows) associated with beef cattle temperament, measured by FS test. Within these windows, six promising candidate genes and their biological functions were identified. These results may contribute to a better comprehension into the genetic control of temperament expression in Nellore cattle.

## Introduction

Cattle temperament is operationally defined as the set of an animal’s behaviors in response to handling by humans, attributing these reactions to general aspects of fearfulness [1, 2]. This concept is closely related to individual differences of reactivity in stressful or challenging situations [3, 4]. There is a growing interest on cattle temperament due to its association with productivity traits, animal welfare and labor safety [5, 6], stimulating cattle producers and researchers to look for a better understanding of genetic and environmental factors that influence the expression of temperament traits, and its impacts on cattle performance and corporate profitability.

Flight speed (FS) test is among the most used behavioral methods to assess beef cattle temperament, reflecting aspects of general fear and agitation [2, 7]. This test was validated in many studies, some of them based on physiological stress indicators [8–10], besides being proved that it is reliable and repeatable in both, the short and long term [11, 12]. Additionally, this method has been described as objective, safe and simple to implement routinely on farm, besides the possibility of integrating this measurement in animal breeding programs requiring minimal additional costs [13–15].

Heritability estimates for FS are moderate to high, ranging from 0.17 ± 0.07 to 0.54 ± 0.16 for *Bos taurus* [14, 15] and *Bos indicus* breeds [16–18]. Additionally, a series of studies have shown favorable genetic correlations of FS with important economic traits, e.g. better temperament (lower FS or higher flight time) was associated with higher average daily gain (ADG) [14, 19], better reproductive performance [20, 21], higher carcass weight and yield [22], and improved meat tenderness [23]. Based on these one can assume that the selection for temperament using FS is feasible and will not produce unfavorable responses on overall performance traits.

Given its phenotypic complexity and, probably, polygenic nature, temperament could potentially be studied using genomic approaches, such as genome-wide association studies (GWAS), in order to identify genomic regions or QTL that affect the underlying genetic architecture related to individual variability [24]. In fact, there were some efforts to identify QTL that account for the variation on beef and dairy cattle temperament. These findings led to several candidate genes distributed throughout the chromosomes, which were associated with a variety of temperament indicators in *Bos taurus* cattle and their crosses, such as: milking temperament [25, 26], isolation temperament and habituation [27], docility [28], flight from feeder and social separation [29], tethering test, weighting test, separation and restraint test [30, 31].

Despite of these initiatives, the use of GWAS to identify candidate regions of the genome associated with temperament of Zebu cattle (and others tropically adapted beef breeds) is still scarce in the literature. We found only two published papers using GWAS for beef cattle temperament, one of them aimed to identify molecular markers associated with temperament at weaning, measured by subjective social separation score in Nellore-Angus crossbred cattle [32]. In the second, a preliminary GWAS study was conducted for FS using BovineSNP50 genotypes in several European crosses (Angus, Hereford, Simmental, Limousin, Charolais, Gelbvieh and Red Angus) [33].

Zebu breeds and their crosses are commonly used for extensive cattle production in warm climates, usually with few contact with humans. Under these conditions, cattle becomes more excitable, impoverishing the animal welfare and increasing risks of labor accidents [5]. Given the importance of assessing cattle temperament and the rapid development of genomic tools, we decided to perform a genome-wide association study aiming to detect genomic regions, potential candidate genes and their biological mechanisms underlying temperament expression (measured by FS test) in Nellore cattle.

## Materials and Methods

### Animals and management

The Committee of Ethical Use of Animals from the Faculty of Agricultural and Veterinary Sciences, São Paulo State University, Jaboticabal—SP, Brazil (Certified n. 0016/14), approved this research.

Data collection was conducted in a private Nellore herd belonging to Agropecuária Jacarezinho Ltda^®^, which has two production units located in the southeast and the northeast regions of Brazil. The field study in both production units was permitted by Dr. Ian Hill, the Chief Executive Officer of Agropecuária Jacarezinho Ltda^®^. The handling procedures were similar in both units. Soon after birth, calves were assigned in handling groups according to the sex, keeping in the same group until weaning (artificially done when the calves were, approximately, 210 days old).

The first performance evaluation is conducted at weaning age, considering weight and visual scores for conformation, finishing precocity and muscling. These data is combined in an index, which is used to define the animals that would stay in the herd after weaning or not (remaining 50% of males and 90% of females). The selected animals are relocated in new handling groups, remaining in the same group from weaning to yearling (approximately 550 days of age), when a second performance evaluation is conducted. This is done by repeating the previous measurements (weaning and scoring each animal for conformation, finishing precocity and muscling), besides measuring scrotal circumference, scoring temperament and assessing breed characteristics. Based on these measurements obtained at weaning and yearling age a new selection index is calculated, and the results are used to define the animals to be culled (50% of males and 10% of females). Breed characteristics and temperament score are not included in the index, however they are assumed as independent criteria, discarding animals with the worst grade for each trait. The pedigree data set included 162,645 animals with 49,597 dams and 1,306 sires. A summary of the data set is shown in Table 1.

**Table 1:** Summary of data set structure and descriptive statistics for flight speed (FS) phenotypes, genotyped animals and pedigree using single-step genomic-BLUP methodology in Nellore cattle.

Number of animals in the relationship matrix	162,645
Number of sires	1,306
Number of dams	49,597
Number of phenotyped animals	16,660
Number of CG^a^	735
Number of SNPs after quality control	455,374
Number of genotyped animals remaining after quality control	1,384

^a^CG, contemporary group.

### Assessment of cattle temperament

Flight speed (FS) test was “devised to measure the time taken for an animal to cover a set distance after leaving a confined area” [17]. In this study the measurements were carried out when each animal left the cattle crush after weighing. The time taken by each animal to cover a known distance (ranging from 1.25 to 3.0 m, according to the facilities design) was recorded with an electronic device, comprising two pairs of photoelectric cells, a stopwatch and a processor. Time and distance were used to calculate FS phenotype, in m/s. The assumption of this test is that higher speed animals have more excitable temperament. The FS records were taken from 16,660 animals that were born between 2008 and 2012. This test was carried out simultaneously with the performance evaluation at yearling, trying to minimize the interference in the farms handling routines. In the present dataset, FS ranged from 0.08 to 6.67 m/s, with an average (± SD) of 2.35 ± 1.04 m/s.

### Genotype information and quality control

A total of 1,405 phenotyped animals were genotyped with high density bead array technology: Illumina Infinium HD Assay^®^ and Illumina HiScan System^®^. The High Density Bovine SNP BeadChip contains 777,962 SNP markers spread across the genome at a mean distance of 3.43 kb between markers. Genotyped animals, born between 2008 and 2009, were randomly selected following the criteria of at least 5 animals in each contemporary group. As part of quality control (QC), the following were excluded: SNP markers with unknown genomic position, located on sex chromosomes, monomorphic and markers with minor allele frequency (MAF) bellow 0.05, call rate bellow 90%, with excess markers heterozygous genotypes and those who had a mean intensity of the low cluster. Individuals with call rate bellow 90% were also excluded. After QC, a total of 455,374 SNPs and 1,384 genotyped animals remained.

### Statistical analysis

All analyzes, to estimate variance components and genetic parameters as well as the genome-wide association study (GWAS), were performed using a single-step procedure (single-step GBLUP—ssGBLUP) which combined, simultaneously, all phenotyped animals, pedigree information and genotypes in one step [34], using Bayesian inference [35] via Gibbs sampling. The animal model for FS included direct additive genetic and residual effects as random, and contemporary groups (CG: farm and year of birth, sex, farm at yearling and management groups at birth, weaning and yearling) as fixed effect. Age of animal at the time of temperament assessment was included as a covariate with linear and quadratic effects. The CG containing less than five animals and records out of the range given by the mean of the CG 3 SD were excluded from the analyses. To perform the statistical analyses, the GIBBS2F90 software was used [36] and the model can be represented in matrix form as:

y=Xβ+Za+e

where is ***y*** the vector of FS observations; is ***β*** the vector of fixed effects; ***a*** is the vector of direct additive genetic effects, assuming $a\;\sim \;N\;\left(0,\;H\sigma_{\text{a}}^{2}\right)$, where is ***H*** the relationship coefficients matrix among animals and $\sigma_{\text{a}}^{2}$ genetic additive variance; ***X*** is the corresponding incidence matrix for the fixed effects; ***Z*** is the incidence matrix of the random additive direct genetic effects (associates ***a*** with vector ***y***); ***e*** is the vector of the residual effect in which $e\;\sim \;N\left(0,\;I\sigma_{e}^{2}\right)$, where ***I*** is the identity matrix and $\sigma_{e}^{2}$ is the residual variance. The *prior* distribution for genetic and residual variance components was an inverted Wishart and the posterior estimates were obtained using the POSTGSF90 program [37].

The inverse of ***H*** matrix was obtained as [38]:

$$H^{-1}=A^{-1}+\left[\begin{array}{cc} 0 & 0\\ 0 & G^{-1}-\;A_{22}^{-1} \end{array}\right]$$

where $A_{22}^{-1}$ is the inverse of numerator relationship matrix for genotyped animals and ***G***^−1^ is the inverse of the genomic relationship matrix. The ***G*** matrix was constructed weighting each SNP effect by its expected variance in an iterative procedure, following [39]:

$$G=ZDZ^{\prime }q$$

In the ***G*** matrix, ***Z*** is the marker incidence matrix containing genotypes (0, 1 or 2) adjusted for allele frequency, ***D*** is a diagonal matrix with the inverse of expected SNP variance (initially ***D = I***), and ***q*** is a weighting factor. The weighting factor was as in Vitezica et al. [40], ensuring that the average diagonal in ***G*** is close to that of ***A***_22_.

Chains of 800,000 iterations were generated (S1 File) and the first 50,000 iterations were discarded. For parameter estimates, samples were kept each 100 cycles, generating chains with 7,500 cycles. Data convergence was checked through graphical analysis, sampled values versus rounds, and using the criteria proposed by Geweke [41], Heidelberger and Welch [42] and, Raftery and Lewis [43] using the R software, with Bayesian Output Analysis (BOA) package from R 2.9.0 software (The R Development Core Team 2009).

The ssGBLUP method has been used to GWAS [44–46], based on the SNP effects obtained using an iterative process, which increases the weight of SNPs with large effects and reduces the weight of SNPs with small effects as presented by Wang et al. [34]:

1. First step: ***D = I***.
2. Calculate ***G*** matrix: ***G*** = ***ZDZ*′*q***.
3. Calculate GEBV for all animals in the data set using ssGBLUP.
4. Convert GEBV to SNP effects: $\hat{u}=\frac{\sigma_{u}^{2}}{\sigma_{a}^{2}}DZ^{\prime }G^{*-1}{\hat{a}}_{g}=DZ\prime {\left[ZDZ\prime \right]}^{-1}{\hat{a}}_{g}$, where $\hat{u}$ is the vector of marker effect and ${\hat{a}}_{g}$ is the GEBV of the animals which were also genotyped.
5. Calculate the weight for each SNP marker: $d_{i}\;=\;{\hat{u}}_{i}^{2}2p_{i}\left(1-p_{i}\right)$ where *i* is the *i*-th SNP.
6. Normalized SNP weight to remain constant the total genetic variance.
7. Exit or Loop to step 2.

According to Habier et al. [47], the use of SNP windows captures the QTL effect better than a single SNP and is important to discriminate effects from statistical noise [48]. The iterative process was repeated three times, from step 2 to 7, and the percentage (%) of total genetic variance explained by *i*-th consecutive SNPs (SNP window) was calculated following Wang et al. [45]:

$$\frac{var_{\left(ai\right)}}{\sigma_{a}^{2}}\times 100\%=\frac{var_{\left(\sum_{j=1}^{10}Z_{j}{û}_{j}\right)}}{\sigma_{a}^{2}}\times \;100\text{\%},$$

where *a*_*i*_ is genetic value of the *i*-th region that consist of 10 consecutive SNPs, $\sigma_{a}^{2}$ is the total genetic variance, ***Z***_*j*_ is vector of gene content of the *j*-th SNP for all individuals, and ${\hat{u}}_{j}$ is marker effect of the *i*-th SNP within the *i*-th region (S2 File).

### Gene prospection

Consecutive SNPs which explained 1% or more of the total genetic variance were considered as genomic window associated to FS. These regions were used to identify positional candidate genes based on the starting and ending coordinates of each window on *Bos taurus* genome view in the Map Viewer tool available at the National Center for Biotechnology Information (NCBI) platform (http://www.ncbi.nlm.nih.gov) UMD 3.1 version. Functional annotations were obtained using Ensembl Genome Browser (http://www.ensembl.org/index.html) and gene networks were explored by the online Database for Annotation, Visualization and Integrated Discovery (DAVID) v6.7 (http://david.abcc.ncifcrf.gov/). The identification of gene pathways and enrichment analysis (Gene Ontology and KEEG pathways) were performed in DAVID website from the Ensembl annotation. Human genes were used as background in pathway and gene network investigation. A Manhattan plot was created using the R package “ggplot2” (S3 File).

## Results and Discussion

**Fig 1:**
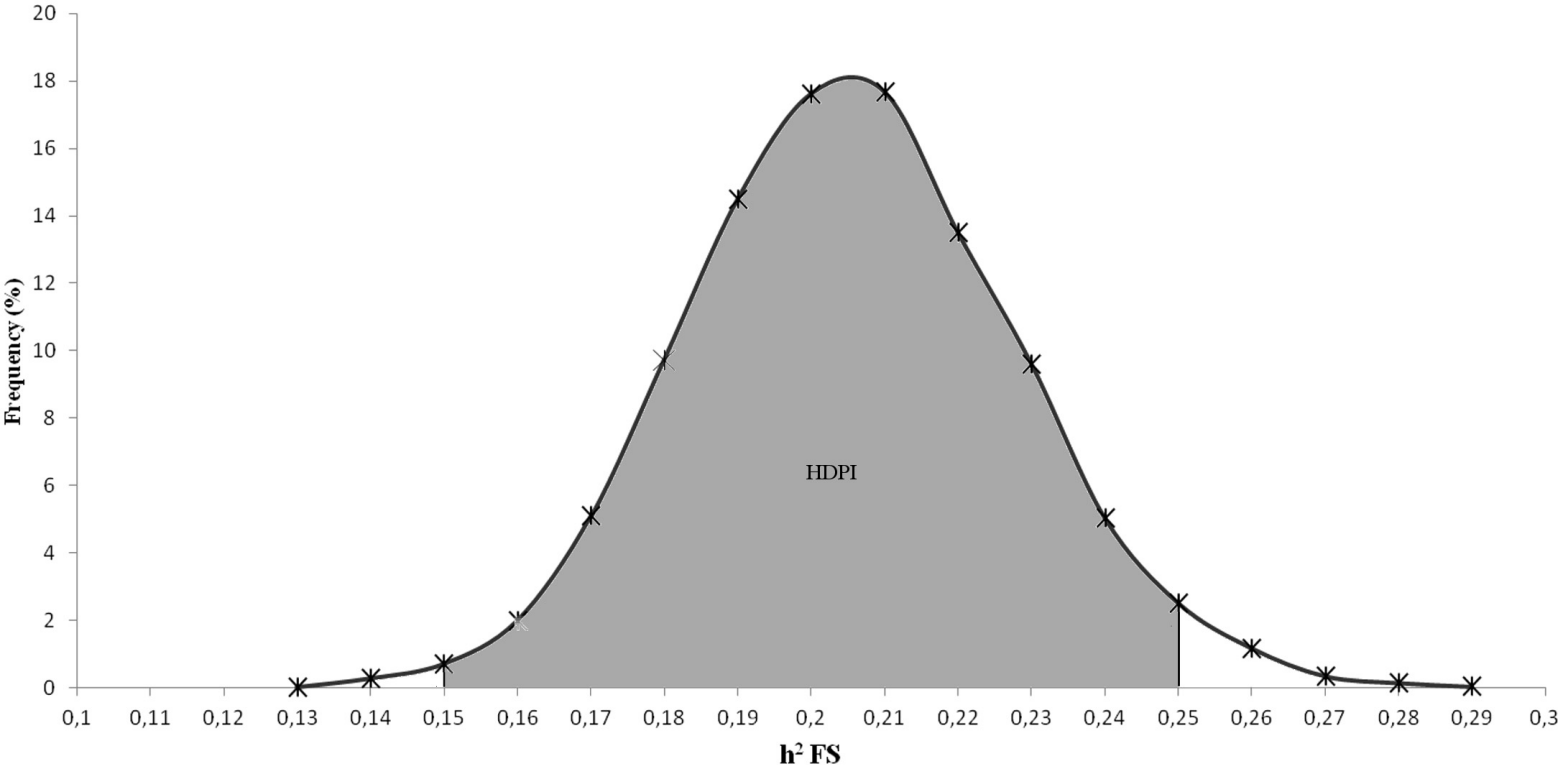
Estimated posterior density of flight speed (FS) heritability in Nellore cattle. The highest probability density interval (HPDI) is presented in gray region.

The convergence criteria used in this study, number of cycles, fixed burn-in period and number of Markov chains were sufficient for convergence of the estimated parameter. Fifty samples were discarded as a fixed burn-in period and the 7,950 interactions remained stable allowing their convergence. Additionally, the convergence was achieved with low SD and a relatively short 95% highest posterior density interval. As a result, the additive genetic ($\sigma_{\text{a}}^{2}$) and residual variances ($\sigma_{\text{e}}^{2}$), and mean of posterior heritability (*h*^2^) for FS were 0.17, 0.65 and 0.21 ± 0.02, respectively. Fig 1 illustrates graphically the posterior distributions of heritability for FS, showing the highest probability density interval (HPDI) that ranged from 0.16 to 0.25.

The heritability estimate (0.21 ± 0.02) is in accordance with the range described in the literature for Nellore and other beef breeds [14, 17, 18, 23] and was similar to that previously presented by our research group using a subset of these data, but not considering genomic information [15, 21]. The additive genetic variability presented by FS indicates the possibility of selecting to improve cattle temperament.

**Fig 2:**
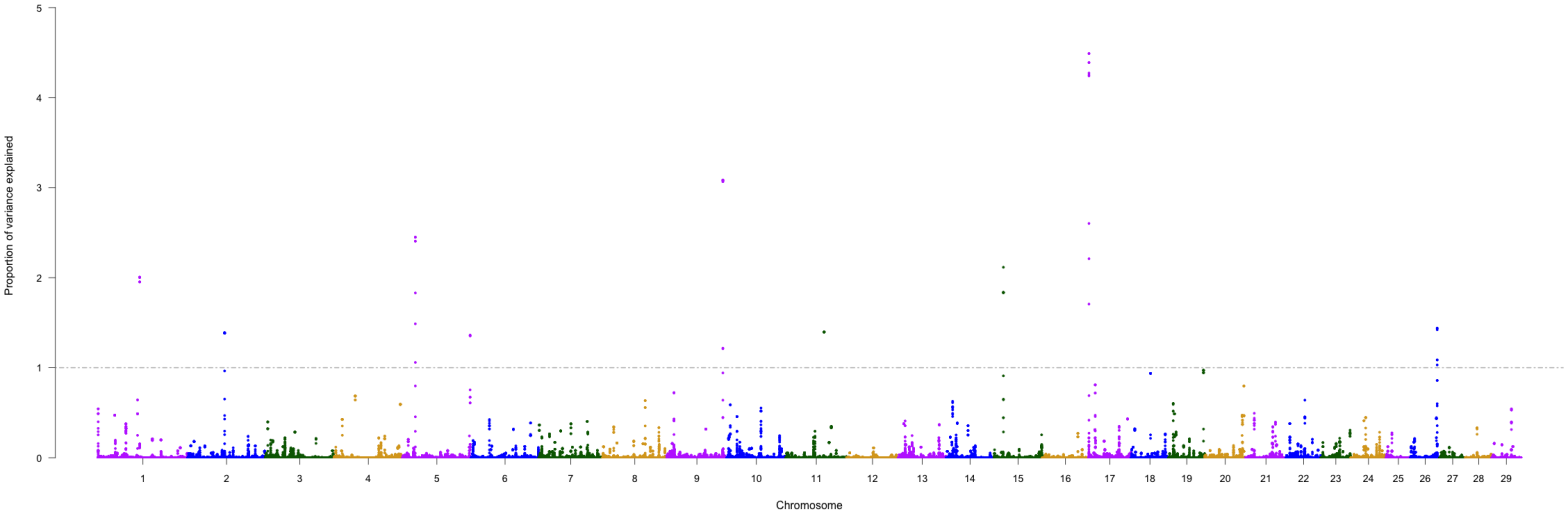
Manhattan plot of additive genetic variance explained by windows of 10 adjacent SNPs for flight speed (FS) in Nellore cattle.

In recent years a large number of potential candidate genes have been associated with several traits of economic importance in farm animals [49, 50]. However, the selection of useful markers for specific traits, such as bovine temperament, should be based on knowledge of the relationship between physiological and/or biochemical process with variation of the phenotypic traits of interest [51]. The additive genetic variance explained by each 10-SNP moving windows is shown in a Manhattan plot (Fig 2). A summary of each SNP window that explained more than 1% of additive genetic variance and the candidate genes is shown in Table 2.

**Table 2:** Summary of SNP windows that explained >1% of genetic variance for flight speed (FS) in Nellore cattle, with a list of annotated genes within each window.

Chr	SNP window^a^		Var (%)^b^	Gene^c^	Gene ID	Gene Location
	Start, bp	Stop, bp				
1	73354330	73406566	2.00	---	---	---
2	65072013	65082628	1.39	*NCKAP5*	786958	64102635–65215462
5	22596661	22604723	2.45	---	---	---
5	119287445	119302785	1.36	---	---	---
9	98759214	98767952	3.08	*PARK2*	530858	98612089–99648791
11	67385287	67404876	1.40	*ANTXR1*	616010	67334564–67590531
15	16598639	16662233	2.12	*GUCY1A2*	613600	16604381–17100655
17	639678	671693	4.49	*CPE*	280753	546402–697925
26	47061401	47095621	1.44	*DOCK1*	537203	46735083–47296206

^a^Windows with 10 consecutive SNPs based on UMD3.1.

^b^Percentage of additive genetic variance explained by each SNP window.

^c^Candidate genes associated to flight speed.

Using the *Bos taurus* genome map, six known genes were found within the significant regions (SNP windows) identified in our study. These were *NCKAP5*, *PARK2*, *ANTXR1*, *GUCY1A2*, *CPE* and *DOCK1* located on the *Bos taurus* chromosomes (BTA) 2, 9, 11, 15, 17 and 26 respectively.

The *CPE* (carboxypeptidase E) gene encodes a multifunctional peripheral protein that plays several non-enzymatic roles in the endocrine tissues and central nervous systems, with the highest concentration found in the brain and pituitary gland [52, 53]. Moreover, it is a prohormone-processing enzyme, which is involved in the biosynthesis of numerous peptide hormones and neurotransmitters [54, 55], including the processing of proinsulin to insulin [56]. The enzyme carboxypeptidase E is responsible for cleaving the C-terminal amino acid residues from peptide intermediates in endocrine cells and neuropeptides in peptidergic neurons [57]. This gene is conserved in different species [53] and polymorphisms in specific regions have been associated with diabetes type 2 and obesity in humans [55, 56]; obesity and infertility in mice [53, 58] and in pigs [59]; with carcass and meat quality in different cattle breeds [51, 60]. The *CPE* expression in the brain is directly influenced by distinct physiological or environmental stress conditions to which animals are exposed, resulting in different levels of hippocampal, cortex and amygdala neuronal degeneration and neuronal survival suggesting a role in neuroprotection [61]. Additionally, this gene was linked to deficient learning and memory [62], modulation of mood and emotional responses [57] in mice.

The functions described above for *CPE* support the correlation estimates presented by Hoppe et al. [14], Sant’Anna et al. [15] and Kadel et al. [22] which reported association of FS with productive traits in beef cattle, such as growth and carcass and meat quality. Additionally, these functions corroborate the relationship of temperament with learning, memory and emotional responses in human [63, 64] and cattle [65]. Thus, *CPE* gene is a potential candidate for future studies aiming to identify specific molecular markers associated with different levels of reactivity in cattle.

The *PARK2* gene, located on BTA9 at 98 Mb, encodes the *parkin* protein, an ubiquitin-protein ligase (E3) which belongs to ubiquitin-proteasome system. According to Hase et al. [66] *PARK2* is expressed in bovine peripheral nerves and brain. These authors showed, by immunohistochemical evidence, that *parkin* is located in bovine axoplasm of myelinated nerve fibers and the Schwann cell outer membrane. The *parkin* protein works as important machinery for intracellular quality control [67–69], marking and degrading misfolded proteins. Mutations in *PARK2* gene affect mitochondrial function and apoptosis in neuronal cells [70], and have been linked with Parkinson [71–73], which is a neurodegenerative disease. Damage in the *parkin* protein function is associated with selective degeneration of dopaminergic neurons [66, 68]. The role of dopamine as a neurotransmitter in central nervous system is well known [74], where its action is mediated by specific receptors to control locomotion, emotional behavior, cognitive functions and memory [75]. Indeed, the dopamine receptor D4 gene (*DRD4*) has been extensively linked to behavior, positive affect and reactivity in mice [76], dogs [77, 78], and horses [79, 80], as well as in humans [81–83] and cattle temperament, measured by a docility test [31].

The *GUCY1A2* gene, located on BTA15 at 16 Mb, encodes an alpha subunit of guanylate cyclase (GC), characterized as guanylate cyclase 1, soluble, alpha 2. The GC is activated by nitric oxide (NO) and catalyzes the conversion of intracellular guanosine-5’-triphosphate (GTP) to cyclic guanosine-3',5'-monophosphate (cGMP), a second messenger in signaling of intracellular transduction pathways [84, 85]. This enzyme has two forms: a membrane protein and a soluble form with specific kinetic properties and tissue distributions [86–88]. The membrane protein consists of a polypeptide chain with a transmembrane receptor which is activated by various endogenous peptides [89] and presents enzymatic activity [84]. The soluble GC (sGC) form is a heterodimeric protein consisting of α (α_1_ and α_2_,) and β (β_1_ and β_2_) subunits encoded by distinct genes [90]. The simultaneous expression of one α- with one β-subunit (α_1_β_1_, α_2_β_1_, α_1_β_2_ and α_2_β_2_ isoforms) is essential for enzyme catalytic activity [91, 92].

The α_2_ subunit, encoded by *GUCY1A2* gene, was firstly identified in human fetal and bovine brain tissues [93]. Subsequently it was isolated in human brain, placenta, spleen, and uterus [94, 95] and in rat brain [96–98]. According to Gibb and Garthwaite [97] and Mergia et al. [98] the highest proportion of α_2_ subunit is found in the mice’s brain, hippocampus and cerebellum. The relevance of this subunit is related to the binding with NO to catalyze the conversion of GTP in cGMP. The signaling pathways NO/sCG/cGMP acts as a phosphodiesterase, ion-gated channel, and cGMP-dependent protein kinases to regulate vasodilation, smooth muscle relaxation, platelet aggregation and neurotransmission [88, 99–102].

The neuronal NO was associated with memory formation as a retrograde messenger. A disruption in exon two of the NO synthase gene increased the aggressive behavior in mice [103, 104]. Additionally, the increase of cGMP level in the brain, with greatest magnitude in the cerebellum, was associated with the occurrence of locomotor activity and depended on the duration of this behavior [105]. Thus, this candidate gene (*GUCY1A2*) could be related to development of the neuronal wiring of cattle temperament. Further studies are required to identify the expression of this gene in bovine brain and the molecular basis associated to highest reactivity (locomotor activity) and poor temperament (expressed by high FS) as well as with vasorelaxation and neurotransmission.

Within the SNP window of BTA26 at 47 Mb, was identified the *DOCK1* gene (dedicator of cytokinesis 1), also known as *DOCK180*, which encodes a member of the dedicator of cytokinesis protein superfamily. In mammalian systems this family consists of eleven members named *DOCK1* to *DOCK11* and classified into four subfamilies, according to the sequence homology [106]. The structure of *DOCK1* contains an N-terminal Src homology 3 (SH3) domain, two Dock homology regions (DHR1 and DHR2 module) and a proline-rich C-terminal region. This protein acts as a guanine nucleotide exchange factor (GEF) for the Rho GTPases (a protein family of small guanosine triphosphates), by signaling the exchange of GDP (guanosine diphosphatase) for free GTP (guanosine-5’–triphosphate). The GEF activity requires binding with engulfment and cell motility 1 protein (ELMO1) [107, 108] to form a bipartite complex, which increases stability for *DOCK1* activity toward GTPase Rac1 [109] in several biological processes, such as endothelial apoptosis [108], cytoskeletal organization [110–112], cell migration and invasion [113–115], tumor cell motility and invasion [116, 117], and phagocytosis [118].

Based on the effects of Elmo1/*DOCK180*/Rac1 signaling cascade on cell migration and proliferation some attempts have been made to study the relationship of *DOCK1* with cancer [117, 119, 120]. Additionally, the effects on migration of neurons [121–123] lead to the association of *DOCK1* with brain development, playing a role in the regulation of axon guidance, dendritic spine morphogenesis in hippocampal neurons [122], axon growth [121] and axon tip motility [124]. Further investigations are important to verify the expression of *DOCK1* gene on the cattle neuronal system and synaptic connectivity and neuronal behavior related to differences in temperament.

Within BTA2 window at 65 Mb the positional known gene is *NCKAP5* (Nck—associated protein 5), which is a protein-coding gene interacting with an SH3 (Src homology region 3) domain of the adaptor protein Nck [125]. Initially, this gene was detected in human brain, leukocytes and fetal fibroblasts [125]. In recent years *NCKAP5* has been associated with human attention deficit hyperactivity disorders [126], bipolar disorders [127], schizophrenia and bipolar disorders [128], susceptibility of primary open angle glaucoma in a Japanese population [129] and increased risk of essential hypersomnia [130] by using GWAS. However, the biological function of this gene is still unclear and future research aiming to clarify the functional analysis and validation are recommended.

Considering the expression of this gene in human brain and leukocytes cells, and the strong association with emotional expression disease, our findings lead us to consider an effect of *NCKAP5* variants on temperament. Further studies are required to identify if this gene is expressed in bovine leukocyte cells. Thus, our result may contribute to elucidate the biological function of *NCKAP5*, considering the influence of brain system on temperament traits [131, 132] and the negative correlation between immune system and stable differences on temperament of mice [133], cattle [10, 134] and infant monkeys [135].

Within the SNP window of BTA11 at 67 Mb, is the *ANTXR1* gene, also known as tumor endothelial marker 8 (TEM8) [136]. This gene encodes a type I transmembrane protein that is recognized as anthrax toxin receptor 1 [137, 138]. No research was found reporting the expression of *ANTXR1* gene in cattle, however it is conserved and expressed in different tissues types of mice and humans [137, 139, 140] and dynamically expressed in chick embryogenesis [141]. The encoded transmembrane protein is a specific receptor of anthrax toxin, which is produced by opportunistic bacteria, *Bacillus anthracis* [138, 142, 143].

A nonsense mutation in *ANTXR1* gene has been associated with human recessive GAPO syndrome [144, 145] which is characterized by a loss of function of TEM8, resulting in growth retardation, alopecia, pseudoanodontia and progressive visual impairment [146]. Moreover, some researchers reported the role of *ANTXR1* in tumor vasculature formation, tumor angiogenesis [147, 148] and regulation of endothelial and fibroblastic activities [149]. *ANTXR1* is within the SNP window of BTA11 and was considered as a candidate gene influencing the expression of FS, however, a possible underlying biological relationship with cattle temperament is still unclear.

The identified regions and positional genes in our study, *NCKAP5* (BTA2), *PARK2* (BTA9), *ANTXR1* (BTA11), *GUCY1A2* (BTA15), *CPE* (BTA17) and *DOCK1* (BTA26) have not been reported in Cattle QTL database [150]. Some research has described QTLs associated with several temperament traits assessed in different cattle breeds and crosses. However, each test considered distinct aspects of this complex trait and the comparison of results should be done carefully. Schmutz et al. [27] evaluated cattle behavior objectively, using an electronic movement measuring device in response to isolation during handling and habituation to the handling procedure. Considering these behavioral traits as a reflection of cattle temperament, the authors reported six associated QTLs on chromosomes 1 (14 cM), 5 (29 cM), 9 (44 cM), 11 (57 cM), 14 (19 and 35 cM) and 15 (12 cM).

On the other hand, Gutierrez-Gil et al. [29] found 29 QTLs distributed across 17 chromosomes affecting temperament traits, which were measured by flight from feeder and a social separation test (scoring to standing alert, vocalization and walking/running), in a Charolais × Holstein cattle population. While Hiendleder et al. [26] classifying dairy cows as ‘nervous/aggressive’ or ‘docile’ during milking, and reported QTLs associated with milking temperament on BTA 5 (136 cM), 18 (105 cM), 29 (20 cM) and X/Y (9 cM).

Additionally, some candidate genes have been linked to beef cattle temperament as result of GWAS using BovineSNP50 [32, 33]. Hulsman Hanna et al. [32] reported a significant association of temperament at weaning, measured in 769 Nellore-Angus crossbred cattle by subjective social separation score, with genes related to sodium ion transport and activity, particularly voltage-gate channel activity, demonstrating individual variability in nervous system responsiveness. Specifically for FS test, Lindholm-Perry et al. [33] performed a preliminary GWAS to discover genetic markers that were associated simultaneously to the variability of feed efficiency (ADG and average daily feed intake) and temperament using 1,057 beef steers of several European crosses (Angus, Hereford, Simmental, Limousin, Charolais, Gelbvieh and Red Angus). These authors reported positional candidate genes on *Bos taurus* chromosome 9 (*quaking—QKI*) and 17 (*glutamate receptor*, *ionotropic*, *AMPA type subunit 2—GRIA2 and glycine receptor β –GLRB*). Interestingly, the gene on BTA 9 reported by Lindholm-Perry et al. [33] is in close proximity (> 1Mb) with *PARK2* gene identified in our study.

Most of the reported chromosome regions and genes, including those identified in the present study correspond to different genome locations. According to Adamczyk et al. [151] the dispersion of bovine genome regions affecting cattle temperament and behavior occurs in function of different methods to assess these traits and specific characteristics of each breed. Furthermore, the identification of regions and positional candidate genes is more accurate using high density marker panels.

An enrichment analysis was performed based on Gene Ontology and their functional pathways to identify a network among the six genes found in our study. However, no interaction among them was identified, demonstrating that each gene is associated with a different metabolic pathway. Among the genes associated with FS expression, greater attention should be given to *CPE*, *PARK2*, *GUCY1A2* and *DOCK1* due to their biological functions related to dopaminergic system, memory formation, biosynthesis of peptide hormone and neurotransmitter and brain development, respectively. In addition, *CPE* is a promising candidate gene which can influence temperament and weight gain simultaneously in cattle due to its function on proinsulin to insulin conversion and the recognized importance of insulin as hormone which modulates feeding behavior in different species [57, 152, 153].

The next step should be the identification of candidate genes associated with other methods used to assess cattle temperament, highlighting their metabolic pathways and potential interactions. This information will provide new insights about the biological mechanisms underlying the expression of this complex phenotype and better understanding about the genetic basis of cattle temperament. Moreover, further efforts are required to validate our findings in other populations in order to generate a DNA test panel for application in marked-assisted selection of Nellore temperament.

## Conclusion

The genome-wide association study presented here allowed us to identify nine regions associated with beef cattle temperament, measured by FS test. Among them, six known genes were identified, contributing to a better comprehension into the genetic control of FS expression in Nellore cattle. In addition, the temperament has sufficient additive genetic variability to respond to selection, being of the same magnitude as other traits traditionally used in breeding programs. Thus, information of markers should help animal selection process in order to improve Nellore cattle temperament and, consequently, the efficiency of production systems.

## Supporting Information

S1 File Gibbs_sampler output. Contain the data used to estimate the genetic parameter for flight speed.
(TXT)

S2 File Percentage of variances. Variance explained by each 10 adjacent non-overlapping SNP window.
(TXT)

S3 File Manhattan data. Contain the data used to generate Manhattan plot for flight speed.
(TXT)

## Abbreviations

SNP: Single nucleotide polymorphism
QTL: Qualitative trait loci
FS: Flight speed
GWAS: Genome-wide association studies
QC: quality control
